# Supplementary material for: Are carbon emissions trading and green financial instruments synergistic? -Comprehensive quantitative research based on content analysis
Source: PLoS One. 2024 Mar 7;19(3):e0298601. doi: 10.1371/journal.pone.0298601 (PMC10919601; doi:10.1371/journal.pone.0298601)
Supplement: S1 Data — (ZIP) [file pone.0298601.s001.zip › Data/地级市Z_F工作报告.zip!/╔╜╬≈/│ñ╓╬╩╨/└┤╫╘╣1⁄2╓┌║┼í╛┬φ┐╦╩2╛▌═°í┐_2018.docx]

各位代表：

　　现在，我代表市人民政府向大会报告工作，请予审议，并请市政协委员和列席人员提出意见。

　　一、2017年工作回顾

　　刚刚过去的一年是长治发展进程中不平凡的一年，我们坚持以习近平新时代中国特色社会主义思想为指引，在市委的坚强领导下，深入贯彻落实新发展理念，全面实施创新驱动、转型升级战略，经济发展和社会事业取得明显成效，在全面建成小康社会的征程中迈出坚实步伐。

　　（一）经济运行重回合理区间。全市地区生产总值完成1477.5亿元、增长7%，自2012年以来首次同步达到全国、全省平均水平。一般公共预算收入完成132.3亿元、增长34.3%，接近历史最高水平。在经济增长重回合理区间的同时，转型发展势头良好，国家高新技术企业由53个增加到68个，战略性新兴产业、高技术产业增加值占工业增加值的比重分别达到4.7%、2.2%。

　　（二）供给侧结构性改革扎实推进。全面完成“三去一降一补”年度任务，关闭3座矿井、退出产能165万吨。加大房地产去库存力度，化解商品房库存297万平方米。多措并举降低企业资产负债率，同比下降1.2个百分点。全面落实降低实体经济成本的各类政策措施，规上工业企业每百元收入成本同比下降4.47元。成为全国首批国家老工业城市和资源型城市转型升级示范区，入选全市域国家光伏发电技术领跑基地。大力推进开发区改革创新发展，5个开发区经省政府批准设立。深入推进国企国资改革，市级党政机关所办69户企业全部与主管部门脱钩，市属37户国有企业完成公司制改革。扎实推进旅游景区体制机制改革创新，18个重点旅游景区实现管理权、经营权“两权分离”。基本完成农村土地承包经营权确权登记颁证工作。潞城市农村集体资产股份权能改革、襄垣县农村集体产权制度改革列为国家试点，长子县闲置凋敝宅基地整治盘活利用、长治县农村集体产权制度改革列为省级试点，推进顺利、成效显现。

　　（三）“三农”工作及脱贫攻坚成效明显。不断优化农业种植结构，调减玉米种植面积11万亩，新发展设施蔬菜3.1万亩，新种植中药材21万亩，成功创建上党中药材全国特色农产品优势区，粮食产量达到16.31亿公斤，创历史新高。大力培育农业经营主体，培育省级示范专业合作社40个，认定家庭农场61个，培训新型职业农民8015人，成功举办首届长治农合发展博览周。加快实施农业品牌战略，新认证“三品”农产品201个，地理标志农产品2个，农产品加工企业销售收入完成217.6亿元、增长6.8%。全力推进脱贫攻坚，全市共有291个贫困村、7.5万贫困人口稳定脱贫，分别超出年初计划的28.2%和10.7%，省级贫困县沁源县摘帽，全市贫困发生率降为3.3%，脱贫攻坚实现再战再胜。

　　（四）城乡面貌变化明显。全面启动“1+3”行政区划调整。全市新建改造市政道路80.2公里，新建改造各类管网1052.2公里，新增城市集中供热面积500万平方米，“三河一渠”综合治理工程基本完工，开工建设207、208、309国道过境段环线、“三馆一区”等项目，启动石子河东西段黑臭水体治理等项目，市政基础设施投资完成37亿元。以“五道五治”为主抓手，全面完成城乡人居环境改善“四大工程”各项任务，建设四好农村路789公里。全市常住人口城镇化率达到52.9%，较上年提高1.4个百分点。实现全国文明城市三连冠，继续蝉联国家卫生城市。

　　（五）生态环境持续改善。严格落实京津冀大气污染“2+26”城市群联防联控措施。加大禁煤力度，全面淘汰主城区20蒸吨及以下燃煤锅炉，各县城淘汰经营性燃煤锅炉197台，完成12.8万户冬季清洁取暖改造。加强综合防控，实行工业企业错峰生产和运输，取缔“散乱污”企业1042个，加大建筑工地、裸露地、道路等扬尘防控治理力度，淘汰黄标车3644辆，实行采暖季机动车单双号限行，完成123家重点排污企业在线监控全覆盖，建立环保举报奖励制度。完成营造林31.5万亩，对全市97条河流分级分段设立“河长”，初步划定2990平方公里生态红线保护区。主城区空气质量综合指数下降8.39%，PM2.5浓度下降13.04%，国控、省控水质断面全面退出劣V类。

　　（六）社会建设和民生保障水平稳步提高。不断加大民生投入，民生支出占全市财政支出的82.6%。积极促进居民增收，城镇居民人均可支配收入突破3万元，农村居民人均可支配收入达到12705元，增速快于城镇居民。扎实办好民生实事，全面铺开12项民生重大工程，超额完成省政府部署的6件民生实事年度任务。优先发展教育事业，完成180所农村寄宿制学校的标准化建设，长子县、长治县通过国务院义务教育发展基本均衡评估验收。努力增加就业，新增城镇就业4.5万人。深入推进医药卫生体制改革，城市公立医院综合改革、县乡医疗卫生机构一体化改革成效明显。全面强化住房保障，完成农村危房改造5142户，建成棚改住房9458套。继续提高城乡低保标准，实现低保线与扶贫线“两线合一”。严格落实安全生产责任，事故起数和死亡人数保持了“双下降”态势。狠抓社会治安综合治理，严厉打击各类违法犯罪，群众的获得感、幸福感、安全感明显增强。

　　（七）政府自身建设不断加强。坚持以党的政治建设为统领，始终同以习近平同志为核心的党中央保持高度一致，扎实开展“两学一做”学习教育常态化制度化、开展维护核心见诸行动主题教育。围绕打造“六最”营商环境，扎实推进“放管服效”改革，取消和下放行政职权事项23项，取消行政审批中介服务11项；积极打造“互联网+政务服务”平台，全年办结公共服务事项42.6万件、增长60%。严格落实中央“八项规定”精神和国务院“约法三章”，加强审计监督，市本级“三公”经费下降19.1%。将重点工作任务列入“13710”电子督办系统，推动各项工作高效落实。自觉接受市人大的法律监督、市政协的民主监督，共办理人大代表建议250件、政协提案383件。

　　各位代表，上述成绩的取得，根本在于习近平新时代中国特色社会主义思想的光辉指引，是省委、省政府正确领导、亲切关怀的结果，是市委统揽全局、科学决策，市人大、市政协有效监督、大力支持的结果，是全市人民齐心协力、奋勇拼搏的结果。在此，我代表市人民政府，向全市人民，向各民主党派、工商联和无党派人士，向各位人大代表、政协委员、离退休老同志，向驻市部队、武警官兵、公安干警，向所有关心支持长治改革发展的各界朋友，表示崇高的敬意和衷心的感谢！

　　在肯定成绩的同时，我们也必须清醒地看到，我市发展不平衡不充分问题十分突出，政府工作仍有诸多问题和不足。具体表现为：一是经济总量不大、结构不优、质量不高，全市GDP总量不足1500亿元，在中部地区87个地市中排在50位以后，低于我市周边的邯郸市、安阳市、焦作市，人均GDP刚刚突破4万元，低于全国平均水平1.7万元；“一煤独大”的结构性矛盾没有根本性改变，煤炭产业依然占到工业增加值的70%，占到GDP的36%，服务业比重仅为42.1%，低于全国平均水平9个百分点、低于全省平均水平11个百分点；319户规模以上工业企业中25%的企业是亏损经营。二是创新驱动不足、科技引领不够，全市研究与试验发展经费投入强度仅为1.17%，只占全国平均水平的一半，每万人发明专利拥有量仅有1.9件，低于全国平均水平近8件，全市高新技术企业数量仅占全省的6%。三是社会事业欠账较多，重大交通基础设施建设不足，农业农村工作基础薄弱，安全生产根基不牢，生态环境问题突出，脱贫攻坚特别是解决深度贫困问题任务艰巨。四是政府职能转变还不到位，部分干部服务意识和法治意识不强，甚至不作为、乱作为，企业对办事慢、办事难反映强烈，等等。我们将以对人民和事业高度负责的态度，以敢于担当、自我革命的勇气，直面矛盾、迎难而上、解放思想、锐意改革，创造无愧于新时代的业绩，决不辜负全市人民的重托。

　　二、2018年工作安排

　　今年是全面贯彻党的十九大精神的开局之年，是改革开放40周年，是决胜全面建成小康社会、实施“十三五”规划承上启下的关键一年。今年政府工作的总体要求是，以习近平新时代中国特色社会主义思想为指导，坚持稳中求进工作总基调，坚持新发展理念，按照高质量发展要求，坚持深化供给侧结构性改革与深化转型综改试验区建设相结合，聚焦“示范区”“排头兵”“新高地”三大目标，统筹推进稳增长、促改革、调结构、惠民生、防风险各项工作，推动质量变革、效率变革、动力变革，在打好防范化解重大风险、精准脱贫、污染防治的攻坚战方面取得扎实进展，加强和改善民生，促进经济社会持续健康发展，努力打造山西重要增长极、建设省域副中心城市。

　　今年发展主要预期目标是：地区生产总值增长7%左右，固定资产投资增长10%，社会消费品零售总额增长7%，一般公共预算收入增长7%，城乡居民人均可支配收入分别增长7%和7%以上，居民消费价格涨幅控制在3%左右，城镇调查失业率、城镇登记失业率分别控制在6%、4.2%以内。

　　主要约束性指标是：万元地区生产总值能耗下降3.2%，万元地区生产总值二氧化碳排放量下降3.9%，万元地区生产总值用水量下降3.9%。环境质量改善指标和主要污染物总量减排指标，完成省下达年度目标任务。

　　各位代表，实现上述目标，任务艰巨、责任重大，我们将进一步坚定信心、鼓足干劲、攻坚克难、全力进取，重点抓好九个方面工作。

　　（一）聚焦“三大目标”，全力抓好转型项目建设。建设资源型经济转型发展示范区、打造能源革命排头兵、构建内陆地区对外开放新高地，是省委、省政府落实习总书记视察山西重要讲话精神、深化供给侧结构性改革、推动经济高质量发展作出的重大部署，转型项目建设是实现“三大目标”的切入点和突破口，我们将切实抓好324个产业转型项目，总投资1786亿元，年度完成投资405亿元。

　　大力培育新兴产业。新兴产业是现代化经济体系最鲜明的特点，是推动经济高质量发展的关键。围绕十大新兴产业发展，重点实施137个项目，总投资1126亿元，年度完成投资223亿元。高端装备制造产业，重点推进经开区潞安ABB智能机器人、襄垣互通科技产业园、高新区际安电气等23个项目，完成投资43亿元；确保立讯精密电子产业园一期、深智精密智能装备制造等项目建成投产。现代煤化工产业，重点推进潞安5万吨乙酸酯、30万吨烯烃分离，潞宝20万吨甲醇、尼龙6聚合等25个项目，完成投资36亿元；确保襄矿乙二醇、潞宝己内酰胺二期等项目建成投产。新能源汽车产业，重点推进武乡镁铝轻量化新材料、沁新集团锂离子电池、清华机械智能排爆车等12个项目，完成投资26亿元；确保成功集团年产30万辆新能源汽车技改项目建成投产。新能源产业，重点推进黎城、平顺500兆瓦领跑基地光伏电站等40个项目，完成投资49.4亿元；确保长子100兆瓦风电、20兆瓦光伏发电等项目建成投产。新材料产业，重点推进高科LED、潞安太阳能电池、高测新型金刚石线等37个项目，完成投资34亿元；确保武乡山予钙业年产10万吨纳米碳酸钙项目建成投产。煤层气产业，重点推进蓝焰武乡区块开采、中联长子区块开采等18个项目，完成投资8亿元；确保200口新井投运产气。现代医药产业，重点推进康宝生物制药、振东创新药物等13个项目，完成投资12亿元；确保康宝血液制品项目建成投产。通用航空产业，重点推进高新区通用航空产业园、屯留航校改扩建、襄垣通用机场等10个项目，完成投资8亿元；确保铱格斯曼飞机清洗液及充气帐篷项目建成投产。节能环保产业，重点推进瑞赛格再生资源循环利用、天元再生资源苯胺等11个项目，完成投资10亿元；确保天脊粉煤燃烧项目建成投产。信息技术产业，重点推进长治遥感卫星地面项目、首创智能产业园等10个项目，完成投资5.8亿元；确保长治工业云项目建成投产。

　　加快改造提升传统产业。围绕信息化和工业化深度融合，以新技术应用、新业态形成、新模式建立为主攻方向，在传统产业领域不断延伸产业链条，走循环发展之路，将断点式生产方式转变为循环式生产方式，提升产品信息技术含量，提高产品供给质量，重点实施69个技改项目，总投资428亿元，年度完成投资150亿元。围绕减量重组、减量置换，提高煤炭先进产能占比，退出煤炭过剩产能165万吨、钢铁过剩产能190万吨，重点推进15个煤矿的提升改造，确保明鑫煤矿、潞安李村煤业、晋煤仙泉煤业等6座矿井投产达效，年度完成投资40亿元。

　　我们将切实把转型项目建设作为“一把手”工程，把项目落地、资金到位、转型项目占比作为考核的主要指标，把开发区作为转型发展的主战场，把招商引资作为干部能力的“试金石”，把深入一线解决问题作为干部的“必修课”，务求项目建设取得更大实效，为加速推动高质量发展、构建现代化经济体系增添新血液、注入新动力，让长治的转型升级之路越走越宽广、越走越自信，让这座资源型老工业城市永葆勃勃生机。

　　（二）聚焦发展短板，全力打好“三大攻坚战”。打好打赢“三大攻坚战”，是全面建成小康社会必须跨越的关口。唯有聚焦聚神聚力，拿出过硬措施，确保风险隐患得到有效控制、脱贫攻坚任务全面完成、生态环境质量总体改善，才能守住长治高质量发展的底线，才能为全面建成小康社会打下坚实基础。

　　有效防范化解重大金融风险。我市金融风险总体可控，但潜在风险和隐患需要高度重视。严厉打击非法集资、金融诈骗等违法活动，依法打击恶意逃废金融债务行为，降低银行不良贷款率，严格规范担保机构、小贷公司等金融机构经营行为，积极防范化解企业债务违约风险和企业担保链风险。持续拓宽企业融资渠道，推动壶化集团在主板上市，加快高科集团、康宝制药、太行紫团上市股改工作，完成天康科技、安得瑞防护等企业在新三板挂牌。着力提高融资规模，做大做强长治银行，加快建设“四大金融平台”，完成沁源、沁县农信社改制。全年社会融资总量达到300亿元以上。

　　持续加大精准脱贫力度。今年我市脱贫攻坚要完成297个贫困村退出、6.46万贫困人口脱贫、武乡和沁县摘帽。切实加强扶贫队伍建设，选派最优秀、最能吃苦、最能打硬仗的同志到脱贫攻坚最前线，扑下身子与贫困群众一起干，攻克艰中之艰、困中之困。集中抓好产业扶贫，启动100万亩中药材种植、300兆瓦光伏扶贫工程，培育100个电商扶贫村、100个旅游扶贫村，发挥农业合作社带动作用，全面推进资产收益扶贫，实现所有贫困村集体经济“破零”。积极推进就业扶贫，增加造林绿化、公益林管护等公益岗位，针对贫困人口开设扶贫车间，对1.4万名建档立卡贫困劳动力开展技能培训，实现稳定就业。扎实推进易地扶贫搬迁，全面开工8897名贫困人口、991名同步人口易地搬迁工程，确保年内竣工率、投资完成率达到60%以上。全面落实政策保障，财政专项扶贫资金增长5%以上，完成金融扶贫小额信贷5亿元以上，落实好教育扶贫资助、“三保险、三救助”政策，稳定推进农村低保和国家扶贫标准“两线合一”，确保贫困群众早日受益，确保脱贫攻坚连战连胜。

　　推进污染防治取得更大成效。我市是京津冀“2+26”联防联控区域通道城市，去年以来不断加大污染防治力度，取得了明显成效，但形势依然严峻，任务十分艰巨。必须持续减少煤炭消费量，在主城区范围内全面禁止燃煤，年内新增集中供热面积1000万平方米，年内建成国化能源调峰储备库、华润燃气应急调峰储配站，保障供气稳定，逐步扩大各县城和城乡结合部的清洁取暖范围。必须持续抓好“散乱污”企业治理，完成重点行业超低排放改造。必须持续加大扬尘、裸地治理力度。必须持续加大重点污染源管控，严厉打击各类环境违法行为。全面实施“河长制”“湖长制”，积极推进沁河、漳河流域水环境综合治理，加强辛安泉域治理保护力度，主城区全面消除黑臭水体，其他县市区黑臭水体消除比例达到80%以上，推进重点经济开发区集中污水处理设施建设。作为资源型老工业城市，工业固废对区域环境影响很大，必须切实加大工业固废资源化综合利用，重点研究推进煤矸石、粉煤灰在道路建设、新型建材等方面的综合利用。启动土壤调查评估及修复项目，完成3个矿山生态修复示范工程建设，深入实施化肥农药零增长行动，有效控制农业面源污染。合理划定城镇开发边界、永久基本农田、生态保护三条红线，切实加强森林植被和湿地等自然资源的保护修复，创建1个森林县城、10个森林乡镇、100个森林村庄，完成营造林20万亩，治理水土保持面积43.7万亩。通过努力，我们要让长治的天更蓝、水更清、地更绿。

　　（三）聚焦增强发展活力，全力推进改革开放。改革开放是推动新时代长治高质量发展的活力源泉，我们将以改革开放40周年为重要契机，加快推进关键领域的重点改革，持续不断地走出去、引进来，向改革要活力、向开放要动力，推动长治改革开放走在全省“第一方阵”。

　　深化政府机构改革。国务院机构改革方案已正式公布，省委、省政府已作出安排部署，我们要严格按照部署要求，严明纪律、统筹兼顾、有序推进，全力保障改革期间工作的连续性和衔接性，保质保量完成机构改革的各项任务。通过改革聚人心、强队伍、提效率，进一步优化政府机构设置和职能设置，构建职责明确、依法高效的政府治理体系。

　　打造“六最”营商环境。不断加大对实体经济的帮扶力度，全面落实简政减税减费政策，清理规范涉企收费，进一步减轻企业负担，创新入企服务常态化机制，建立协调会议制度，及时收集汇总企业存在的突出问题和难题，按职责权限全力解决，真正让企业轻装上阵，心无旁骛抓生产、抓经营、抓项目。全面推行企业投资项目审批承诺制，全面实施市场准入负面清单制度，进一步简化审批、优化流程，变先批后建为先建后验，变事前审批为事中事后监管服务。深入推进“证照分离”改革，各类证能减则减，推行企业登记全程电子化，力争全市新登记市场主体数量增长10%。全面推行“双随机、一公开”监管。深入推进“互联网+政务服务”，全面推行网上审批，使更多事项在网上办理，加强政府信息系统互联互通，打通信息孤岛，让数据“多跑路”、群众“少跑腿”，做到“只进一扇门”、“最多跑一次”。积极推进综合执法机构机制改革，着力解决多头多层次重复执法问题。切实为市场主体添活力，为人民群众增便利。

　　深化国企国资改革。加大“僵尸企业”处置力度，制定市属国有僵尸、空壳企业及低效无效资产清理处置实施办法，妥善做好人员分流安置，依法处置债权债务。积极推进国有企业分离办社会职能。完成驻地16户中央企业、17户省属企业“三供一业”分离移交。加快推进市交通燃料公司等12户企业改革。完成61户脱钩企业清产核资。对产业相近、业务相关的企业进行整合重组，增强国有资本竞争力。

　　健全民营经济促进机制。民营企业不仅是我市经济发展的生力军，也是财税增收的重要来源，更是扩大就业的主渠道，创造着半壁江山的GDP，贡献着40%的税收，吸纳着80%的就业，是推动全市转型发展的重要力量。认真落实支持非公有制经济发展政策，实施市场准入负面清单，破除各种隐性障碍。构建“亲”“清”政商关系，健全企业家参与涉企政策制定机制。加强民营经济运行监测，重点监测300个民营企业，妥善应对金融信贷等风险挑战，创建省级“专精特新”企业15户，新增“小升规”企业12户以上，促进各类市场主体平稳健康发展。继续办好“长清班”，打造新时代长治优秀企业家队伍。

　　深化电力体制改革。重点推进襄垣经开区、潞安集团增量配电试点项目。加大直供电力度，将企业直供电范围扩大到60户以上，交易电量突破50亿度。

　　创新用地保障机制。全面开展第三次土地调查。积极推行用地新机制，持续加大拆旧区土地复垦工作，推进城乡建设用地增减挂钩，新增城乡建设用地增减挂钩用地3300亩。加强建设用地项目占用耕地补充，大力推进土地整治和高标准农田建设，新增高等级耕地2650亩。严格落实节约集约用地政策，有效盘活低效闲置土地资源，确保万元地区生产总值用地量下降5.59%。

　　提升对外开放水平。畅通对外开放通道，加快太焦高铁、长治机场改扩建，确保长临高速今年8月份全线通车。开辟长治机场新航线，新增厦门、贵阳等6条航线，北京航班增加至每日3班。开通长治经晋城至郑州城际公交，推进长治至邯郸城际公交，每20分钟一班，票价优惠，法定节假日免费，既为方便全市人民出行，也为吸引更多游客到长治观光旅游、更多客商来长治投资兴业。力争开工黎霍高速，做好平遥经沁源至安泽、壶关经陵川至修武高速公路前期工作。建设对外开放平台，积极谋划长治机场航空口岸临时开放，推进长子能交投铁路口岸开放，年内建成海关及出入境检验检疫综合服务大楼。促进外向型经济发展，扩大优势产品出口规模，壮大出口主体队伍，新培育10家进出口潜力企业，力争出口贸易、实际利用外资分别增长3%。

　　加大招商引资力度。把长治市招商引资奖励办法当作抓好招商工作的“梧桐树”，精心谋划包装招商引资项目，以开发区为重要招商载体，面向京津冀、长三角、珠三角、环渤海、中原城市群等区域，主动对接大企业、大集团，组织开展专场推介活动，进行一对一、点对点的精准招商、以企招商、以商招商，确保年内每个县市区至少引进落地5个以上、建成投产3个以上重大产业转型项目，全市招商引资完成项目签约2050亿元，当年签约项目的开工率达到30%以上。

　　（四）聚焦动能转换，全力实施创新驱动战略。发展是第一要务，创新是第一动力，人才是第一资源。我们必须在强化科技引领、发挥人才作用、提升质量水平上下实功、出实招，加速推动长治高质量发展新旧动能转换。

　　加大科技创新力度。积极争创国家创新型城市试点，重点推进与中科院半导体研究所、海外华人高新技术协会共建产学研合作平台，充分利用各类科创平台集中开展联合攻关，催发新技术、新产品、新业态，全年新建2个科技创新平台，新增2个省级以上企业技术中心、15家高新技术企业，把握创新发展主动权。设立5000万元科技成果转化引导基金，推动科研成果转化应用，全年技术合同交易额达到10亿元。加大发明专利奖励力度，确保有效发明专利拥有量突破680件，发明专利申请量达到350件。

　　促进“双创”上水平。推广唯美诺“创意+工厂”、易淘“电商+创业”等模式，鼓励发展多形式“双创”载体，重点推进潞城市城南双创基地、双创—长轴1945老工业区产业文化园、长锻双创基地等项目，打造“双创”体系产业链条。新培育省级双创基地2家、省级双创示范基地1家，力争创建国家级小微企业双创示范基地1家。

　　强化人才支撑。提高引进人才政策标准，提供500套拎包入住的人才公寓，加大新设立院士、博士工作站奖励力度，吸引更多高层次人才来长治发展。发挥长治职业技术教育资源优势，加强校企合作，加大产教融合，推进高技能人才培训基地、技能大师工作室建设，培育一批精益求精独具特色的长治工匠，培养600名高技能人才。全面落实以增加知识价值为导向的分配政策，完善科创人员创新激励政策，采取更加灵活的薪酬制度和奖励措施，真正激发长治科创人员的积极性和创造力。

　　推进质量强市。紧抓我省被批准为国家标准化综合改革试点省的机遇，大力实施标准化战略，积极争创全国质量强市示范城市。深入开展企业质量提升行动，培育10家以上质量提升标杆企业。加大品牌培育力度，组织企业参加山西品牌“中华行、丝路行、网上行”活动，新培育10个山西省名牌产品、2个地理标志保护产品。

　　（五）聚焦二十字方针，全力实施乡村振兴战略。实施乡村振兴战略是新时代“三农”工作的总抓手。我们要以更大的决心、更明确的目标、更有力的举措推动农业全面升级、农村全面进步、农民全面发展，谱写新时代长治乡村振兴新篇章。

　　科学制定战略规划。按照“产业兴旺、生态宜居、乡风文明、治理有效、生活富裕”的总要求，出台长治乡村振兴战略规划（2018至2022年）和“5+1”六个专项规划，坚持农民主体地位、乡村全面振兴，因地制宜、循序渐进，科学谋划推进乡村振兴的重大项目、重大改革、重大举措，突出基础设施建设、基本公共服务体系、支撑政策体系，为我市全面实施乡村振兴战略描绘蓝图、明确方向、指明路径。

　　大力发展有机旱作农业。创建全国绿色有机旱作农业示范市，是贯彻落实习总书记山西视察“要坚持走有机旱作农业的路子”重要指示精神的重大举措，是省委、省政府赋予我市的重大使命，我们要全力推进、务求实效。今年重点围绕耕地质量提升、农水集约增效、旱作良种攻关、农技集成创新、农机配套融合和绿色循环发展六大工程，创建12个3000亩以上的有机旱作农业封闭示范区，生产纯天然、高质量的农产品。积极推进旱作农业标准化生产和品牌创建，建设100万亩标准化生产示范基地，新认证“三品一标”农产品90个，继续举办好第二届长治农合发展博览周，打造“长治神谷”等20个区域公共品牌、企业品牌和产品品牌，推进上党中药材、太行有机小米特色农产品优势区建设。

　　深入推进农业现代化。进一步优化农业种植结构，减少玉米种植面积5万亩，新增特色农产品种植面积10万亩。做大做强龙头企业，重点扶持太行紫团、沁州黄小米等100个农业产业化骨干企业，大力推进长清生物80万吨玉米深加工、汇禾源小杂粮加工等100个农业产业化项目建设，不断完善“公司+合作社+基地+农户”生产经营模式，培育省市示范合作社80个，新认定家庭农场100个，加快建设3个省级现代农业产业园、10个市级现代农业产业园，推动农村一二三产业深度融合，带动农业全环节升级、全链条增值。深度开发党参、连翘茶等功能食品和药食同源产品，促进产业融合。加大农业科技创新，推进“互联网+现代农业”，提高农业科技水平、提升特色农产品竞争力。

　　深化农村综合改革。以“三权分置”重大理论创新为指导，衔接落实好第二轮土地承包到期后再延长30年的政策。全面完成农村承包地确权登记颁证，促进土地有序流转。积极开展农村土地经营权抵押贷款试点工作。完成襄垣县、长治县改革试点任务，全面铺开农村集体产权制度改革。全面实施农村闲置凋敝宅基地和闲置农房整治盘活利用。深化林权、水权、供销社改革，激活农业农村生机和活力。

　　推进美丽宜居乡村建设。以农村垃圾、污水治理和村容村貌提升为重点，实施农村人居环境集中整治三年行动计划，统筹解决好农村道路、饮水、信息通讯、教育、医疗等公共服务设施。加快推进四好农村路建设，新建改建农村公路500公里。新启动12个省级、20个市级和100个县级美丽宜居示范村。加强传统村落保护，继承发扬乡村优秀历史文化，促进农村移风易俗，不断健全自治、法治、德治相结合的乡村治理体系。通过努力，要把我们的乡村刻画成现代文明与田园乡村交相辉映，看得见山、望得见水、记得住乡愁的新图景。

　　（六）聚焦新型城镇化建设，全力抓好主城区建设和现代服务业发展。

　　建设现代文明生态宜居城市。按照“拉大框架、扩容提质、多规合一”要求，高标准做好城市总体规划修编，精心编制滨湖新区、太焦高铁站、长丰地块等十大重点片区的控制性详规，实现主城区范围控规全覆盖，年内完成“1+3”行政区划调整工作。重点推进十大城建工程：1.筹资30亿元，推进207、208、309国道过境段环线工程建设，力争主线完工，彻底改变国道干线穿城而过的历史。2.加快推进总投资27亿元的“三馆一区”建设，年内完成主体工程。3.按照滨湖新区规划，严格管控整治周边建筑，加大漳泽湖水污染治理，环绕漳泽湖湿地建设步行、慢行、骑行、车行、绿行“五行”系统，开辟休闲健身新空间，打造魅力长治新名片。4.启动高铁站站前广场及相关配套设施建设，年内建成高铁站到机场的快速通道，把高铁站片区打造成为我市集现代交通枢纽、现代物流集散、现代服务业集聚于一体的城市靓丽新地标。5.开展主城区架空线路专项整治，今年重点整治10条主干道、20条次干道和50条巷道的“蜘蛛网”。6.开工建设总投资24.4亿元的新长北干线、保宁门街改造等7条市政道路工程，同步改造各类管网425公里，年内建成通车。7.开工建设八一桥等3座市政桥梁和长治学院等2座人行过街天桥，新建改造20座城市公共厕所，新增2000个城市停车位。8.建成投资2.5亿元的紫金西街、五针街铁路立交桥和襄垣城际连接线工程，改扩建长治南高速出口，疏通城市出入通道。9.积极推进生活垃圾分类处理，开工建设投资5亿元的垃圾发电项目，加快实施污泥餐厨垃圾处置项目，新建10座垃圾中转站，在各级党政机关、居民区、学校、医院、宾馆、城中村等选取50个试点开展分类示范工作，推进垃圾减量化、资源化、无害化处理。10.加快推进棚户区、城中村改造，推进已开工的5000套棚改住房建设，确保年底交付使用，围绕长丰片区、天晚集片区等集中连片棚户区新开工建设4578套棚改住房；加快实施主城区11个城中村改造工程，完成马坊头、史家庄等6个村庄改造。我们还将切实加大园林绿化工作的力度，新增绿地面积200万平方米，整治裸地150万平方米，完成泽馨公园建设，国庆节向市民全面开放。各县市区都要积极围绕主城区扩容提质，实施一批重点城建工程，全面提升县城综合承载能力，加快推进与主城区交通设施互通互联、公共服务均等共享、产业发展高度融合，推动上党城镇群建设提档次、出精品、上水平，使长治真正成为山西的省域副中心城市。

　　大力发展现代服务业。加快构建立体式现代物流格局，重点推进黎城智慧能源物流港、长治烟草物流配送中心等14个项目建设，完成投资22.9亿元。加快发展现代商贸业，重点推进万达广场、经开区现代物流园等10个项目建设，完成投资6.2亿元。大力发展电子商务，新增1个电子商务示范县、2个电商产业园，培育5家市级电商示范企业，新改扩建500个村级电商服务站，覆盖率超过70%。力争全市服务业增加值占GDP比重提高1个百分点。

　　（七）聚焦太行板块，全力抓好文化旅游产业发展。长治山川壮美、历史悠久、文化灿烂，是八百里太行最美地段，大美太行在长治。我们要建设全国一流、国际知名的全域旅游目的地，加快把文化旅游产业培育成我市新的战略性支柱产业。

　　打造太行旅游品牌。坚持高标准规划，编制以太行板块为主的全域旅游发展规划。大力解决旅游交通瓶颈问题，开工建设总投资54亿元、全长289公里的太行一号旅游专用线，构建四通八达的旅游交通网络，同步谋划沿线旅游资源的深度开发。深入挖掘红色旅游资源，大力发展红色旅游，推进黎城黄崖洞兵工厂等景区景点的提档升级，把上党革命老区打造成为全国红色旅游目的地。发挥市文旅集团龙头作用，加快推进全域旅游发展。引进战略投资者，重点推进神龙湾、太行欢乐谷等项目建设，新建改建旅游厕所129座，力争太行山大峡谷成功创建国家5A级景区。围绕建设全国休闲农业和乡村旅游示范市，促进旅游与农业、文化、康养等产业深度融合，重点提升培优20个乡村旅游精品示范点，扶持40个“农家乐”开展标准化建设，重点打造推介10条乡村旅游精品线路和100个精品景点。强化旅游市场综合监管，优化旅游服务环境。综合利用多种方式加大旅游宣传力度，举办好太行旅游养生体验季系列活动。把太行旅游打造成为世界知名品牌，吸引全国及世界各地游客来长治欣赏山水太行、体验文化太行、品味绝美太行。

　　促进文化惠民。牢固树立社会主义核心价值观，大力弘扬太行精神，不断巩固扩大全国文明城市创建成果，拓展群众性精神文明创建活动，深入实施公民道德建设工程，选树长治好人、道德模范、感动人物、文明家庭，培育良好家风、民风、社风，切实提高长治文明程度。强化国家公共文化服务体系示范区建设，提升公共文化服务水平，继续办好周末大剧院、流动图书车、农村公益电影放映、送戏下乡、上党文化大讲堂等文化惠民活动。加快推进四个院团的排练场地建设，确保年内投入使用。

　　加快文化产业发展。运用文化产业普查成果，支持大中小微多层次文化企业发展，积极发展文化创意等新业态，推动文化产业与旅游、体育、金融、科技等融合发展，着力打造一批市场化的文化产业基地。加强文化遗产和文物保护利用，加大文化传承保护与开发力度，创作更多文艺精品。

　　（八）聚焦提升群众获得感，全力保障民生需求。不忘初心，人民至上。我们必须始终把人民对美好生活的向往作为奋斗目标，时刻放在心头、抓在手上，持之以恒为群众办实事、解难事，让改革发展成果更多更公平惠及长治人民。

　　优先发展教育事业。以提升我市高考升学率为切入点和突破口，强化队伍管理、加大考核奖励，确保我市高考升学率一年有改观、两年见成效、三年大提升。实施学前教育第三期行动计划，确保学前三年毛入园率达到91%以上。加快推进主城区8所义务教育学校建设，确保长子门小学、火炬中学改扩建等5所学校建成投入使用。推动县域内城乡义务教育一体化发展，完成165所农村寄宿制学校标准化建设，确保所有县通过国务院义务教育发展基本均衡评估验收。加强教育信息化建设，促进教育管理和教学质量提升。支持特殊教育、民办教育、职业教育、继续教育健康发展。强化师德师风建设，办好人民满意的教育。

　　促进就业增收。按照“人人持证、技能社会”的要求，实施全民技能提升工程，对5万名农村转移劳动力、城镇失业人员、企业在岗职工进行职业技能培训，并取得相应技能等级证书，积极举办各类职业技能大赛，提高劳动者就业能力。统筹做好高校毕业生、农村转移劳动力、城镇就业困难人员、退役军人、残疾人等重点群体就业工作，帮助就业困难人员实现稳定就业，确保零就业家庭动态清零。深入推进农民工工资无欠薪市县创建活动，坚决打击恶意拖欠农民工工资行为，严格落实最低工资标准，保障居民收入持续增长。

　　加快建设健康长治。深化县乡医疗卫生机构一体化改革，进一步完善“三医”联动改革机制，推动医改工作走在全省前列。健全完善分级诊疗制度，做好家庭医生签约服务。加快推进市医院、市二院综合楼、市妇幼保健院改扩建等项目建设。鼓励规范社会办医，加快发展中医药事业，积极筹建市中医院综合楼项目。深入开展爱国卫生运动。积极推进医养结合。推动全民健身和全民健康深度融合，促进体育事业发展，加快体校建设，积极备战山西省第十五届运动会，做好第二届全国青年运动会赛事筹办工作。

　　加强社会保障体系建设。今年在财力十分紧张的情况下，各级政府都要保持对社会保障投入的稳定增长，扎实办好省政府“六件民生实事”。再次提高城乡居民基本养老保险基础养老金财政补助标准，每人每月由85元提高到108元，继续提高城乡居民基本医疗保险财政补助标准，每人每年由450元提高到490元，健全被征地农民基本养老保险制度。实施社会保障全覆盖计划，促进全民参保登记，逐步消除社会保障覆盖盲点，实现人人享有基本社会保障。

　　（九）聚焦发展底线，全力维护社会安全稳定。安全稳定是人民美好生活的基础和前提。我们必须牢固树立总体国家安全观，坚决守住“三条底线”，确保人民安居乐业、社会安定有序。

　　切实抓好安全生产。坚持“四铁”要求，严格落实各项措施。严格企业履行安全生产主体责任，确保安全责任、投入、培训、管理、应急救援“五到位”。全面推进安全风险分级管控和隐患排查治理“双防控”体系建设，提高隐患源头治理能力。扎实推进安全生产责任体系、全民安全素质提升、安全生产执法检查长效机制“三项建设”，稳步提高政府安全监管效能和企业安全管理水平。持续深化煤矿、非煤矿山、道路交通、危化品、建筑施工、燃气、特种设备等重点行业领域专项整治，切实减少一般事故，有效遏制较大事故，坚决杜绝重特大事故，确保全市安全生产形势保持稳定。

　　深化平安长治建设。以创建全省最安全城市为目标，扎实推进社会治安防控“六网覆盖”工程，严厉打击违法犯罪活动，深入开展扫黑除恶专项斗争。持续加大毒品打击力度，开展“雷霆扫毒”专项行动，从严打击吸毒贩毒违法行为，坚决遏制毒品来源，严格强制戒毒。提高预警、应急和依法处置能力，严防大规模群体性事件、突发环境事件、重大食品药品安全事故、重大交通事故等公共安全事件发生。强化极端天气应对，做好防灾减灾救灾工作。

　　加强社会综合治理。坚持共建共治共享，推进社区、社会组织、社会工作“三社联动”，推动社会治理重心向基层下移。做好社区矫正工作，加强对特殊人群的帮扶和管理。扎实做好信访工作，维护和解决好群众合理利益诉求，坚决打击以访谋利、组织非访等违法行为，不断提高社会治理水平。

　　切实加强统计、科普、档案、地方志、人民防空、双拥共建工作，发展老龄、慈善和红十字会事业，支持工会、共青团、妇联、残联等群团组织改革发展，做好民族宗教、外事侨务、对台各项工作，支持国防建设和军队改革，为全市改革发展汇聚社会各界正能量和强大合力。

　　各位代表，新时代对政府工作提出新要求，各级政府及其工作人员一定要坚持在党的领导下，牢固树立“四个意识”，坚定“四个自信”，以时不我待的紧迫感、夙夜在公的使命感，扎实开展“不忘初心、牢记使命”主题教育，加强学习、强化管理，促进思想观念大转变、本领素质大提升、工作方法大改进，不断提升政府履职能力和服务水平。

　　一要勤政为民。人民政府为人民，全心全意为人民服务是我们的天职。全市各级政府要牢固树立以人民为中心的发展思想，始终以人民对美好生活的向往为奋斗目标，以造福人民为最大政绩，时刻把群众的安危冷暖放在心上，想群众之所想，急群众之所急，多谋民生之利，多解民生之忧。我们要严格执行人大及其常委会的决议决定，认真办理人大代表建议、政协委员提案，依法接受人大监督，自觉接受人民政协的民主监督，主动接受司法监督、群众监督和舆论监督。广泛听取各民主党派、工商联、无党派人士和人民团体意见。全面推进依法行政，坚持科学、民主、依法决策，努力建设人民满意的政府。

　　二要务实担当。新时代赋予了我们加快长治发展、造福上党人民的历史重任，我们要以昂扬向上、奋发有为的精神状态，迎难而上、知难而进，问题面前不回避、困难面前不推脱、逆境面前不退缩，工作在一线落实、问题在一线解决、作风在一线转变，真正做到守土有责、守土负责、守土尽责。落实容错纠错机制，旗帜鲜明地褒奖、鼓励担当者，支持、保护担当者；完善问责机制，旗帜鲜明地对不作为、慢作为、乱作为行为严肃问责，让务实担当精神在上党大地蔚然成风。

　　三要高效服务。改革发展，服务至上。我们要刀刃向内、自我革命，不断提升全市政府系统的服务水平，提高全体工作人员改革创新、狠抓落实、驾驭风险的本领，对企业诉求、项目需求、群众期盼，认真回应、主动解决，不说不能办，只讲怎么办，真正为企业、为群众提供优质高效服务。全面深化、拓展、延伸“13710”信息化督办制度，切实强化政务督查，全力破除“中梗阻”，全面打通“最后一公里”，推动政府各项工作见人见事、抓紧抓实、抓出成效。

　　四要廉洁从政。廉洁为从政之本。我们要深入推进政府系统党风廉政建设，坚持把纪律和规矩挺在前面，严格执行中央八项规定精神及实施细则，紧盯形式主义、官僚主义新表现，坚持不懈纠“四风”、正作风。完善财政预算管理，严控“三公”经费，加强审计监督。全面推进政务公开，让权力在阳光下运行。全市政府工作人员要廉洁修身、勤勉尽责，干干净净为人民做事，决不辜负人民公仆的称号。

　　各位代表，上党大地人杰地灵，长治人民勤劳睿智。这片土地铸就了伟大的太行精神，创造过世人瞩目的奇迹和辉煌。走进新时代，我们都是建设者、开创者、见证者。让我们高举习近平新时代中国特色社会主义思想伟大旗帜，在省委、省政府和市委的坚强领导下，不忘初心、牢记使命，为把长治打造成为山西重要增长极和省域副中心城市，为实现长治经济更加繁荣、社会更加和谐、生态更加美丽、人民更加富裕而努力奋斗！
